# PHD-2/HIF-1α axis mediates doxorubicin-induced angiogenesis in SH-SY5Y neuroblastoma microenvironment: a potential survival mechanism

**DOI:** 10.1038/s41598-025-89884-3

**Published:** 2025-03-03

**Authors:** Ahmed M. Abou-Shanab, Ola A. Gaser, Noha Galal, Alaa Mohamed, Dina Atta, Samaa Samir Kamar, Shireen Magdy, Mennatallah A. Khedr, Hoda Elkhenany, Nagwa El-Badri

**Affiliations:** 1 Center of Excellence for Stem Cells and Regenerative Medicine, Zewail City of Science and Technology, Giza, 12578 Egypt; 2 Biomedical Sciences Program, University of Science and Technology, Zewail City of Science and Technology, Giza, 12578 Egypt; 3 Histology Department, Kasr Al-Ainy Faculty of Medicine, Cairo University, Cairo, Egypt; 4 Department of Surgery, Faculty of Veterinary Medicine, Alexandria University, Alexandria, 22785 Egypt

**Keywords:** Tumor microenvironment, Neuroblastoma angiogenesis, Doxorubicin, Neo-angiogenesis, PHD-2/HIF-1α targeting, Cancer microenvironment, Cancer therapy, Embryonal neoplasms, Oncogenes, Paediatric cancer, Tumour angiogenesis, Cancer, Chemical biology

## Abstract

The response of neuroblastoma (NB) cells to chemotherapeutics and their influence on NB microenvironment remain incompletely understood. Herein, we examined the underlying molecular mechanism via which Doxorubicin, a chemotherapeutic agent used for NB treatment, promotes proangiogenic response in the SH-SY5Y microenvironment. Doxorubicin treatment at 1 µg/ml reduced SH-SY5Y cell proliferation and primed the apoptosis pathway. Unexpectedly, SH-SY5Y cells treated with doxorubicin upregulated their expression of the pro-angiogenic factors, including vascular endothelial growth factor (VEGF), platelets-derived growth factor (PDGF), and matrix metalloprotease-2 (MMP-2) and secretion of nitric oxide. To assess the functional angiogenesis of SH-SY5Y cells pre-treated with doxorubicin, an indirect co-culture system with human umbilical vein endothelial cells (HUVEC) was established. These HUVECs acquired enhanced proliferation, migration capacity, and tube formation capability and exhibited increased nitric oxide (NO) production, in addition to upregulated α-smooth muscle actin expression, suggesting enhanced contractility. In-ovo studies of the neo-angiogenic response of SH-SY5Y pre-treated with doxorubicin further show their promoted neo-angiogenesis as indicated by the generated blood vessels and histological analysis of CD31 expression. Inhibition of PHD-2 could be a potential target for doxorubicin, as indicated by molecular docking, molecular dynamics (MD) simulation, and MM-GBSA calculations, leading to hypoxia-inducible factor-1 alpha (HIF-1α) stabilization. Bioinformatics analyses and enrichment analyses of RNA-seq data revealed activation of Pi3K pathway which is further validated in-vitro. These results provide evidence of the unexpected pro-angiogenic response of SH-SY5Y cells to doxorubicin treatment and suggest the potential use of multi-modal therapeutic regimens for a more comprehensive approach to NB treatment.

## Introduction

Neuroblastoma (NB) is considered the most occurring extracranial solid tumor during infancy and childhood. Nearly 90% of the patients at the diagnostic stage are less than 5 years old while only less than 5% of patients are adults^1^. NB originates mainly in the neural crest cells during the development of the peripheral sympathetic nervous system because of genetic and epigenetic alterations that lead to impaired differentiation of sympathetic neurons^2^. Accordingly, the site of the NB mainly represents the final destination of the migrating neural crest cells and their progeny which in most cases will be either the adrenal medulla or paraspinal ganglia^3,4^. The heterogenicity of the tumor highly influences the clinical outcomes and the prognosis of NB and demonstrates a broad-spectrum range from spontaneously regressive tumor with no therapeutic intervention to relentlessly aggressive metastatic tumor^5,6^. NB microenvironment also plays a pivotal role in tumor growth, progression, and therapeutic resistance. Now, there are many approaches through which the characterization of the tumor microenvironment (TME) can be achieved such as single-cell transcriptomic analyses which allow transcriptomic profiling of various cell populations that can be validated by their sorting and isolations using FACS followed by in-vitro studies^7^. In a study by Dong et al. of 16 tumors (2 ganglioneuroblastoma and 14 neuroblastomas) from untreated patients, various cell populations were recognized such as non-immune cells, including fibroblasts, Schwann cells, endothelial cells, and immune cells, such as myeloid cells, dendritic cells, and T and B cells^8^. Further studies on neuroblastic tumors also reported the infiltration of various immune cells such as low cytotoxicity natural killers (NK), immunosuppressive myeloid cells, dendritic cells, and dysfunctional T cells^9–11^.

Tumor growth depends on a suitable blood supply via the formation of endothelial microvessels in which pro-angiogenic factors outweigh the anti-angiogenic factors. This phenomenon, known as the angiogenic switch, recruits endothelial precursor cells to form new blood vessels^12,13^. For instance, Pezzolo et al. reported that NB tumor cells expressing Oct-4 and tenascin C (TNC) could be considered progenitors for tumor-derived endothelial cells that might contribute to tumor growth and chemoresistance^14^. Earlier in 1999, Rössler et al. showed that the expression of vascular endothelial growth factor (VEGF), an important proangiogenic factor, is upregulated by the hypoxic microenvironment of NB resulting in enhancing tumor angiogenesis by paracrine mechanisms^15^. Later, analyses from datasets of tumor gene expression of 88 neuroblastoma samples revealed high expression of both hypoxia-inducible factor-1A (HIF-1α) and hypoxia-inducible factor-2α (HIF-2α) but not HIF-3α^16^. In a study by Dungwa et al., HIF-1α was upregulated in malignant neuroblastomas but not benign tumors and it contributed to the tumor’s necrotic core and aggressiveness^17^. Moreover, it was reported that overexpression of HIF-1α in NB leads to activation of sonic hedgehog (SHH) signaling which subsequently increases tumor angiogenesis, migration, and invasiveness^18,19^. However, other studies demonstrated that HIF-2α is a critical factor that influences the expression of VEGF protein and is associated with poor clinical outcomes and prognosis of NB^19–21^. In response to hypoxia, tumor cells adapt by changing the transcription of genes involved in angiogenesis, cell survival, and metabolism. HIF-1α and HIF-2α are critical for this adaptive response^22^. In the presence of oxygen, prolyl hydroxylases (PHDs) modify HIF-α proteins at two conserved prolines, resulting in HIF interaction with the von Hippel-Lindau (VHL)-E3 ligase protein complex, targeting HIFs for ubiquitylation and subsequent proteasomal degradation^23–25^. Thus, suppression of PHD-2 aids HIFs stability and functionality promoting tumor proliferation and metabolism, as well as limiting reactive oxygen species (ROS) levels^26–28^. Interestingly, HIF-1α was suggested to preferentially target` glycolytic enzymes supporting tumor growth^29,30^.

Doxorubicin is frequently used as a chemotherapeutic, but it was reported to possess various side effects, including induction of pro-inflammatory and pro-angiogenic responses within tumors. For instance, adenosine 2B receptor blocking attenuates the effect of etoposide and doxorubicin in increasing the expression of VEGF and interleukin-8 (IL-8) in human melanoma cell line^31^. Treatment of a mutant p53 cell line with doxorubicin induces the pro-metastatic sphingolipid enzyme SK1, which stimulates a tumor intrinsic promigratory response and an extrinsic pro-angiogenic response mediated by p38/MAPK activation, BMP4 production, and stabilization of the transcription factor Snail^32^. NB samples obtained from early necrotic regions were associated with higher microvascular density. The same group also hypothesized that changes related to chemo-resistance development included an enhanced pro-angiogenic activity, demonstrating, both in-vitro and in-vivo, a shift to a pro-angiogenic phenotype in chemo-resistant tumors^33,34^. Moreover, metronomic cisplatin is found to promote tumor angiogenesis and tumor growth via increased mobilization of proangiogenic BMDCs at certain low doses^35^. In another study, HIF-1α was upregulated in tumor cells in response to doxorubicin enhancing VEGF secretion and synthesis of NO, stimulating tumor angiogenesis^36^. In NB, chemotherapeutic drugs display pro-angiogenic effects such as cisplatin, doxorubicin, and vincristine which showed an increase in tube formation and sprouting of endothelial cells with higher microvessel density through promoting the level of cell kinases phosphorylation including angiogenesis-related kinases such as protein kinase Cβ and Akt^37^.

Herein, we aim to investigate the effect of doxorubicin treatment on SH-SY5Y NB cell line proliferation, apoptosis, and pro-angiogenic response. We also studied the PHD2/HIF axis as a potential target for DOX.

## Results

### Doxorubicin treatment alters SH-SY5Y morphology and inhibits their proliferation

**Fig. 1:**
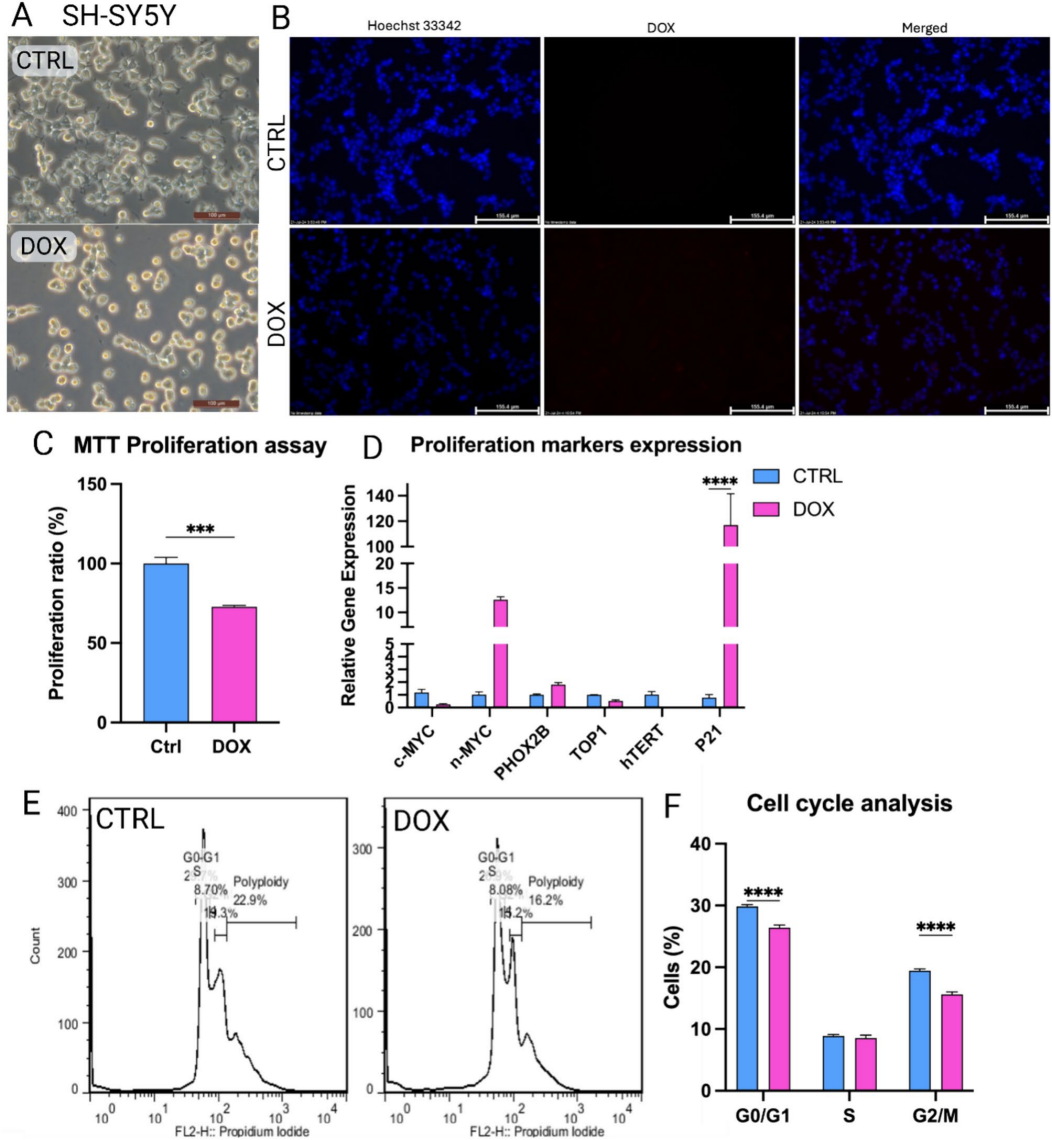
Doxorubicin (1 µg/ml) inhibits SH-SY5Y NB cell proliferation. (**A**) Morphological assessment of SH-SY5Y in both CTRL and DOX groups. (**B**) Fluorescence analysis of doxorubicin localization on the nucleus. (**C**) MTT proliferation analysis of SH-SY5Y. (**D**) Quantitative analysis of proliferation markers in SH-SY5Y cells upon doxorubicin treatment. (**E**) Flow cytometry analysis of cell cycle phases. (**F**) Bar graph representation of the difference between cell populations in different cell cycle phases. *p*-values: * *p* < 0.05, ** *p* < 0.01, *** *p* < 0.001 and **** *p* < 0.0001.

Microscopic analysis showed distinct morphological changes in SH-SY5Y cells following doxorubicin treatment at a concentration of 1 µg/ml compared to the CTRL group (Fig. 1A). CTRL SH-SY5Y cells displayed the characteristic elongated or bipolar morphology with prominent neurites, thin thread-like extensions essential for neuronal function. The cytoplasm appeared homogenous with evenly distributed organelles. Conversely, doxorubicin-treated cells exhibited a significant alteration in shape, becoming more spherical, rounded, and shrunken. Neurite retraction and membrane blebs were evident, suggesting potential impairment of cellular communication and doxorubicin-induced apoptosis.

Further, the cellular uptake of doxorubicin at a concentration of 1 µg/ml was assessed. Fluorescence microscopy showed co-localization of doxorubicin within nuclear DNA stained with Hoechst 33,342 dye in the DOX group (Fig. 1B). This confirms doxorubicin reaching its target site within the nucleus.

MTT assay demonstrated a significant decrease in cell proliferation in the DOX group compared to the CTRL group at concentrations of 1, 10, 50, and 100 µg/ml while treatment at 0.1 µg/ml did not show any effect (Supplementary File 1, Fig. 5). Figure 1C suggesting that doxorubicin treatment at a concentration of 1 µg/ml effectively disrupts SH-SY5Y cell growth.

Figure 1D shows no alterations in the mRNA expression levels of oncogenic drivers MYCN, PHOX2B, and C-MYC, nor cell cycle regulators TOP1 and hTERT (Fig. 1D). Interestingly, the expression of cell cycle inhibitor p21 was significantly upregulated in DOX group.

Flow cytometry analysis of the cell cycle distribution revealed a significant decrease in the G0/G1 and G2/M cycle phases in DOX group compared to CTRL (Fig. 1E, F). The S phase population remained unchanged. This suggests that doxorubicin contributes to the observed decrease in proliferation through altering cell cycle progression.

### Doxorubicin treatment induces apoptosis in SH-SY5Y cells

**Fig. 2:**
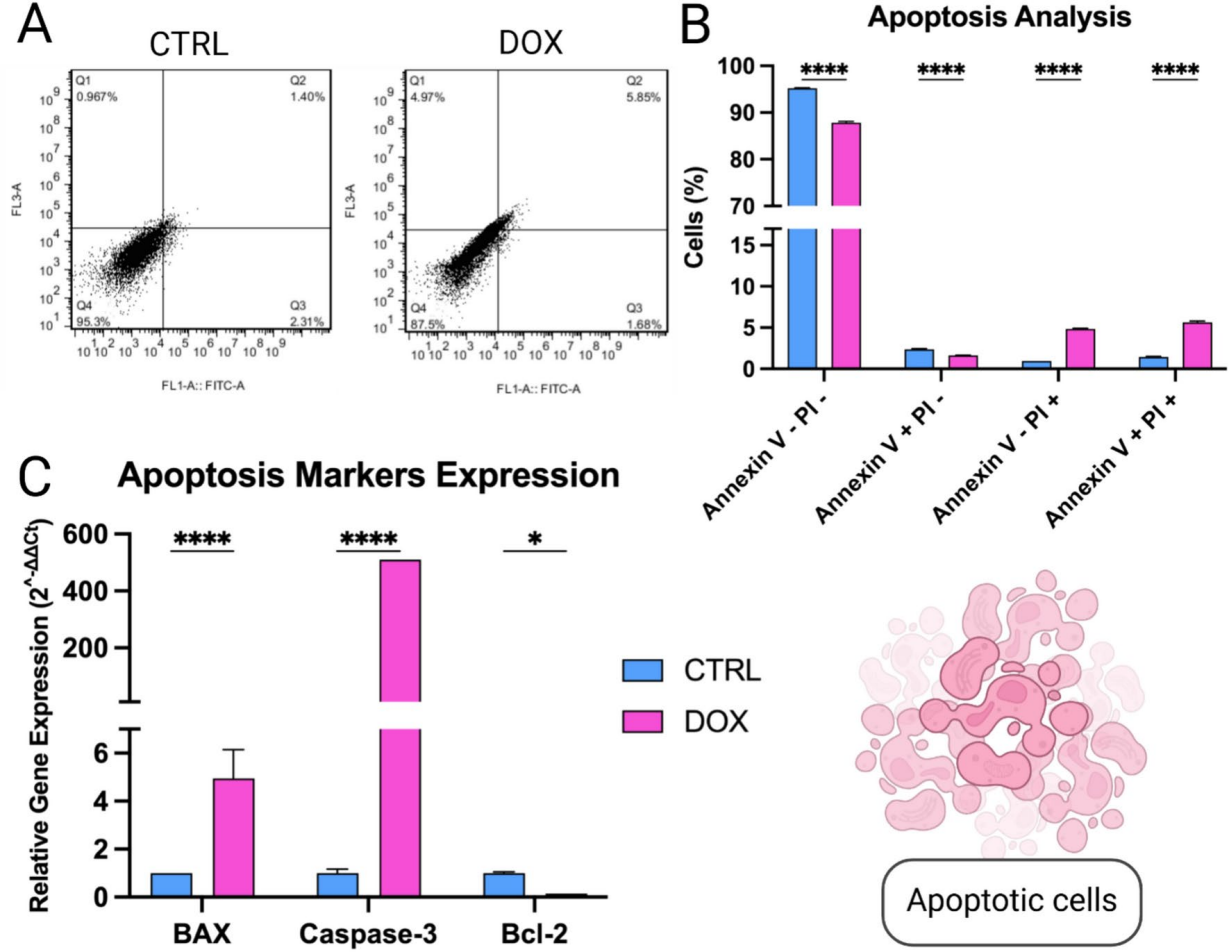
Apoptotic induction in SH-SY5Y after treatment with doxorubicin (1 µg/ml). (**A**) Flow cytometry analysis of Annexin V/PI staining. (**B**) Bar graph representation of the cell populations in early apoptosis, late apoptosis, and necrosis. (**C**) Quantitative analysis of apoptotic markers using qPCR. *p*-values: * *p* < 0.05, ** *p* < 0.01, *** *p* < 0.001 and **** *p* < 0.0001.

Annexin V/PI staining was employed to quantify apoptosis induction by doxorubicin treatment at a concentration of 1 µg/ml (Fig. 2A). Analysis showed a significant decrease in the early apoptotic population (Annexin V + /PI-) in DOX-treated cells compared to the CTRL (Fig. 2B). Conversely, the late apoptotic population (Annexin V+/PI+ showed a significant increase in the DOX group compared to CTRL (Fig. 2B). This indicates a substantial induction of late-stage apoptosis by doxorubicin. Additionally, doxorubicin treatment resulted in a significant increase in the necrotic cell population (Annexin V−/PI+ compared to CTRL (Fig. 2B).

To further explore the molecular mechanisms of apoptosis induction, qPCR was performed to assess the expression of key apoptotic markers (Fig. 2C). We observed a significant upregulation of pro-apoptotic markers BAX and Caspase-3 in doxorubicin-treated cells. Interestingly, the expression of the anti-apoptotic marker Bcl-2 is downregulated. These findings suggest that doxorubicin triggers apoptosis in SH-SY5Y cells primarily by activating the BAX/Caspase-3 pathway.

### Doxorubicin treatment upregulates pro-angiogenic paracrine factor secretions in SH-SY5Y niche

**Fig. 3:**
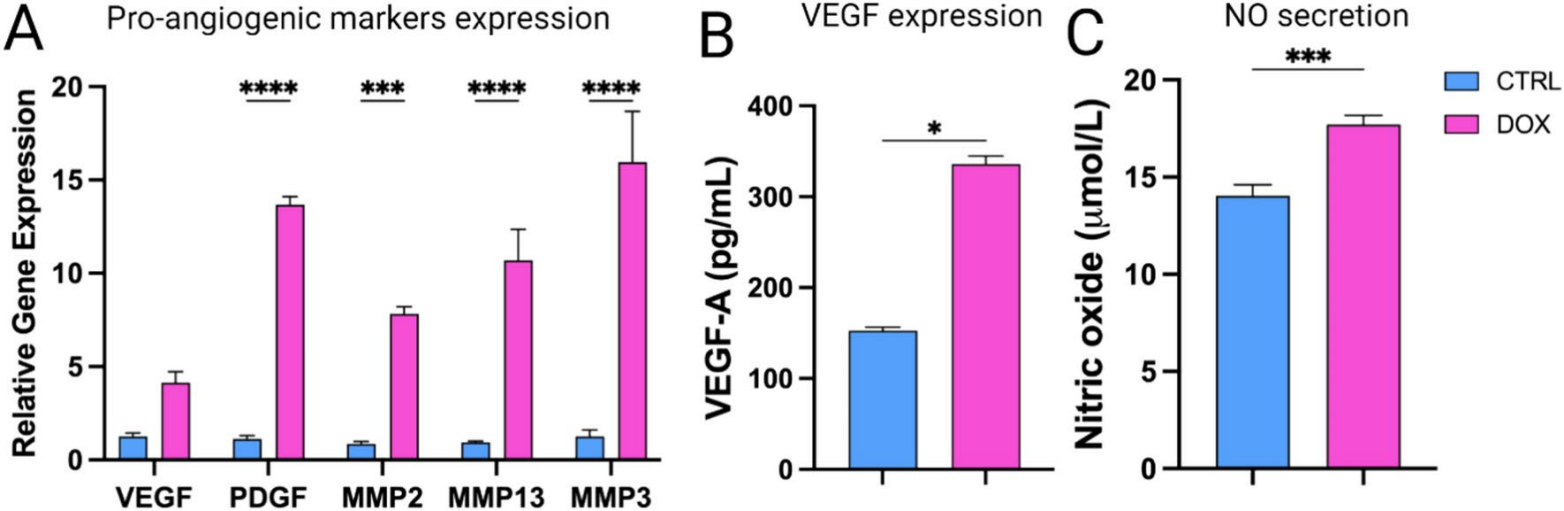
Doxorubicin (1 µg/ml) induces SH-SY5Y cells’ secretion of pro-angiogenic factors. (**A**) Genotypic expression of pro-angiogenic factors expression. (**B**) Quantitative analysis of VEGF protein expression using ELISA assay. (**C**) Measurement of NO production in SH-SY5Y cells. *p*-values: * *p* < 0.05, ** *p* < 0.01, *** *p* < 0.001 and **** *p* < 0.0001.

Our data showed a significant upregulation in the mRNA expression of pro-angiogenic factors, including VEGF, PDGF, MMP-2, MMP-13, and MMP-3 (Fig. 3A). This suggests that doxorubicin treatment at a concentration of 1 µg/ml may induce the production of factors that promote blood vessel formation and ECM remodeling. To confirm the protein level changes for key angiogenesis factors, an ELISA analysis was performed on VEGF. We observed a significant increase in VEGF protein secretion in DOX group compared to the CTRL (Fig. 3B). This finding supports the mRNA data and suggests a potential pro-angiogenic response by the SH-SY5Y cells upon doxorubicin treatment.

Finally, our data showed a significant increase in signaling molecule NO secretion in DOX group compared to CTRL (Fig. 3C).

### Doxorubicin-treated SH-SY5Y cells promote HUVEC vasculogenesis

**Fig. 4:**
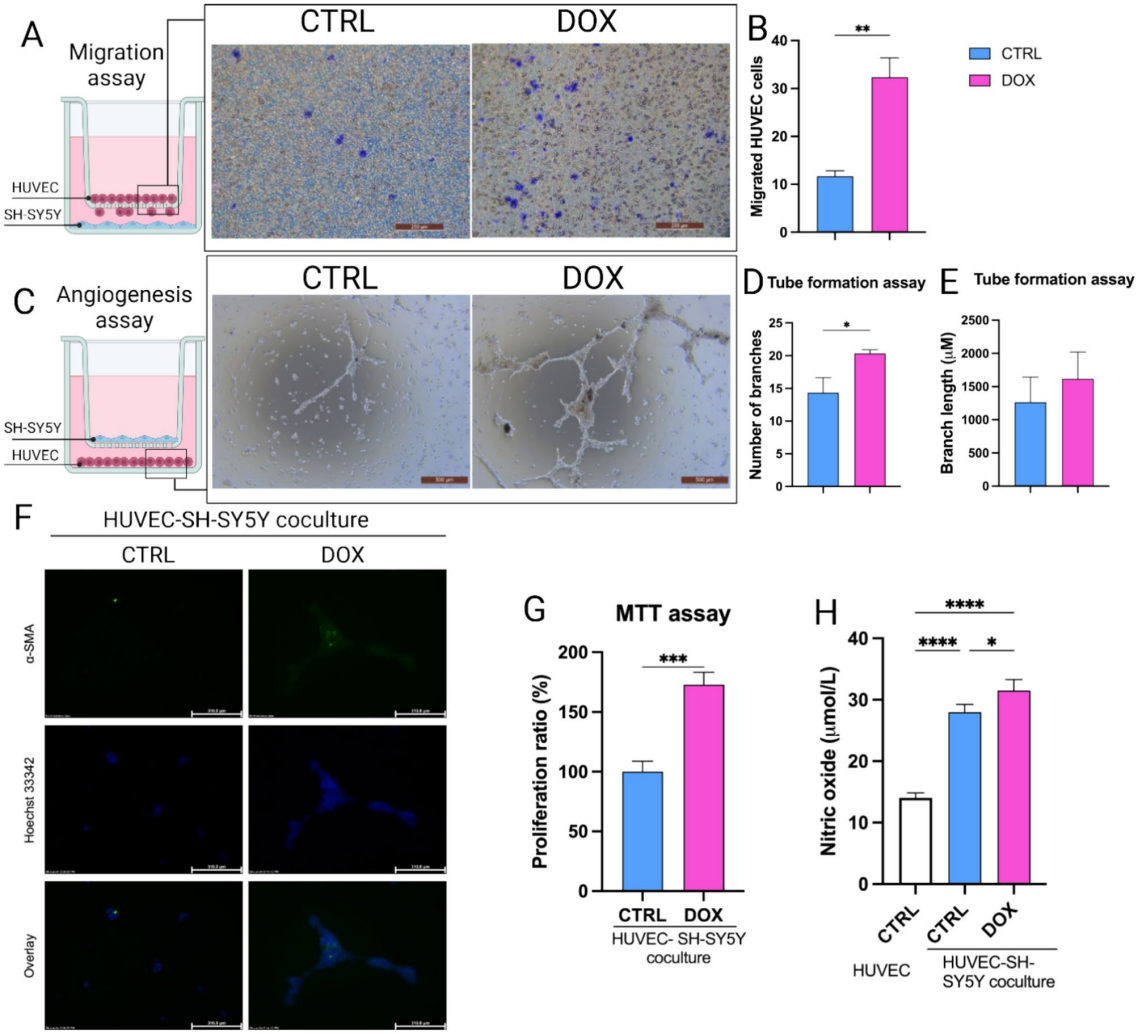
Assessment of in-vitro HUVEC vasculogenesis upon co-culture with doxorubicin-treated SH-SY5Y cells (1 µg/ml). (**A**) Migration assay of HUVECs. (**B**) Bar graph representation of the number of migrating HUVECs. (**C**) In-vitro HUVEC tube formation assay. (**D**) Bar graph representation of the number of the generated branches. (**E**) Bar representation of the length of the generated branches. (**F**) Immunostaining of α-SMA in HUVEC. (**G**) MTT assay to assess HUVEC proliferation. (**H**) Quantification of NO secretion by HUVEC. *p*-values: * *p* < 0.05, ** *p* < 0.01, *** *p* < 0.001 and **** *p* < 0.0001.

To assess the functional impact of doxorubicin-mediated paracrine factor secretion in SH-SY5Y niche, we investigated HUVEC migration and vasculogenesis in an in-vitro co-culture system (Fig. 4). Transwell migration assays (Fig. 4A) revealed a significant increase in HUVEC migration towards doxorubicin-treated SH-SY5Y cells (at a concentration of 1 µg/ml) compared to those co-cultured with CTRL SH-SY5Y cells (Fig. 4B). This suggests that doxorubicin treatment stimulates the secretion of factors that promote HUVEC migration, potentially contributing to angiogenesis.

To evaluate the influence of SH-SY5Y cells on HUVEC vasculogenesis, they were indirectly co-cultured with either SH-SY5Y cells from CTRL or DOX groups (Fig. 4C). The number of branching structures formed by HUVECs was significantly higher when co-cultured with doxorubicin-treated SH-SY5Y cells compared to the CTRL group (Fig. 4D). This indicates that doxorubicin treatment enhances the ability of SH-SY5Y cells to support HUVEC network formation, a key step in vasculogenesis. Interestingly, the length of these branches was not significantly affected by doxorubicin treatment (Fig. 4E).

To corroborate the observed increase in HUVEC migration, we analyzed the expression of α-SMA, a marker of contractile endothelial cells. HUVECs co-cultured with doxorubicin-treated SH-SY5Y cells (at a concentration of 1 µg/ml) exhibited a significant upregulation of α-SMA compared to those co-cultured with CTRL SH-SY5Y cells (Fig. 4F). This suggests that doxorubicin treatment promotes a more motile and potentially more mature endothelial cell phenotype. Finally, MTT assay revealed a significant increase in HUVEC proliferation when co-cultured with doxorubicin-treated SH-SY5Y cells compared to CTRL (Fig. 4G).

To further explore the angiogenic response, we assessed NO secretion by HUVECs, which plays a complex role in their angiogenesis. A significant upregulation in NO secretion was observed in HUVECs co-cultured with doxorubicin-treated SH-SY5Y cells compared to the CTRL (Fig. 4H).

### Stimulated neoangiogenesis of doxorubicin-treated SH-SY5Y in-ovo

**Fig. 5:**
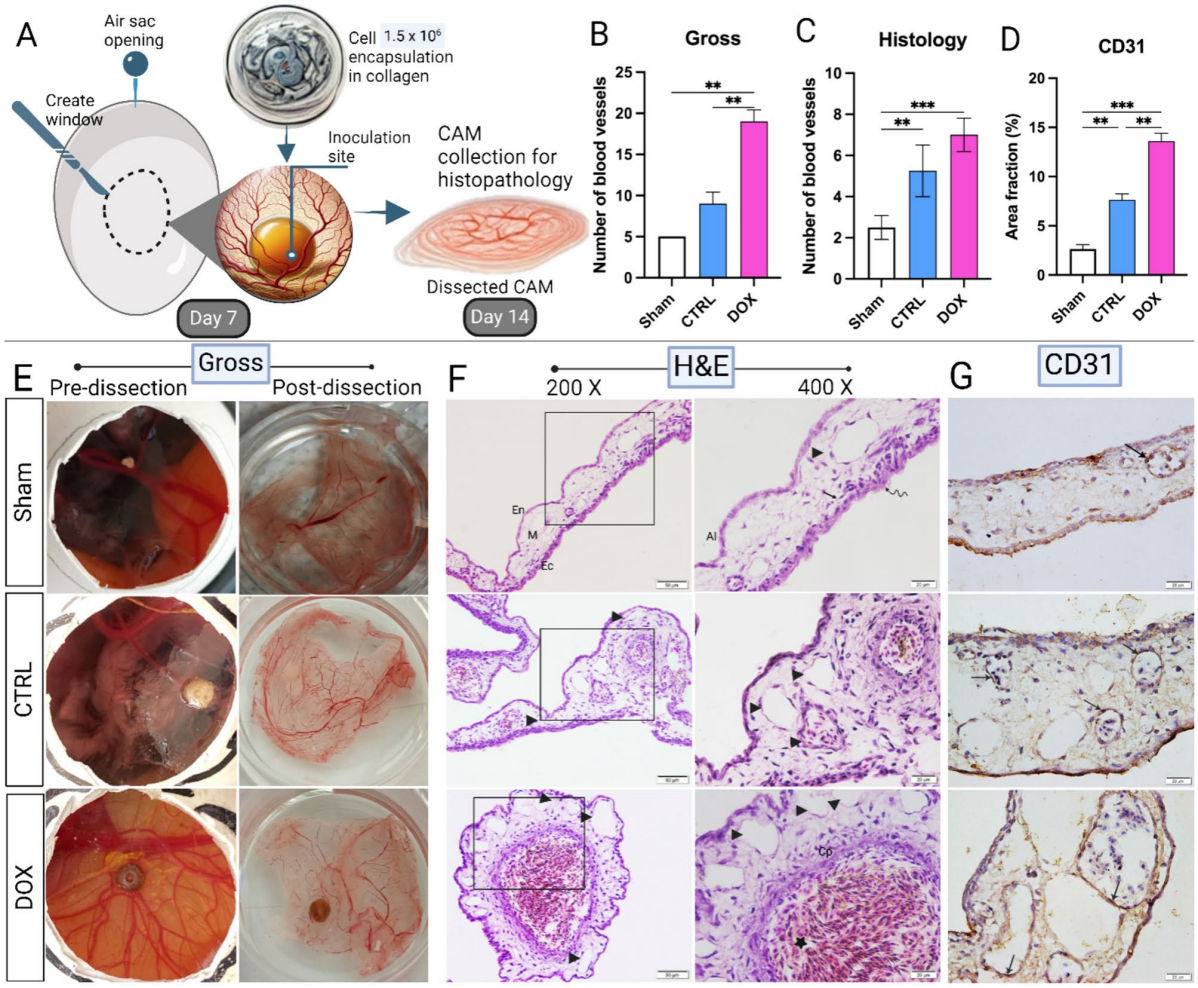
Doxorubicin treatment (1 µg/ml) enhances SH-SY5Y-induced neo-angiogenesis in-ovo. (**A**) Workflow of SH-SY5Y engraftment on CAM. (**B**) Bar graph representation of the number of the newly generated blood vessels in-ovo. (**C**) Histological analysis of the newly formed blood vessels. (**D**) CD31 immunohistochemistry staining analysis. (**E**) In-ovo analysis of neo-angiogenesis induced by SH-SY5Y cells. (**F**) Histological analysis of CAM from different treatment groups. (**G**) Immunohistochemistry analysis of CD31 expression to mark the endothelial progenitor cells. *p*-values: * *p* < 0.05, ** *p* < 0.01, *** *p* < 0.001 and **** *p* < 0.0001.

Figure 4A illustrates the workflow for investigating the neo-angiogenesis of SH-SY5Y in-ovo to further confirm the effect of doxorubicin (Fig. 5A). Upon SH-SY5Y encapsulation in collagen matrix and seeding onto the CAM, cells were incubated for 7 days. On day 14, SH-SY5Y cells pre-treated with doxorubicin treatment at a concentration of 1 µg/ml showed substantial blood vessel formation (Fig. 5E), and more extensive branching (Fig. 5B).

Histological analysis of the CAM sections in the Sham group shows that chick embryo CAM at day 14 displays three layers: the ectoderm (Ec) showing the chorionic epithelium (wavy arrow) and subchorionic capillary sinus (arrow), the mesoderm (M) containing blood vessels (arrowhead), and endoderm (En) showing the allantoic epithelium (Al) (Fig. 5F). In CTRL group, the mesoderm shows the generation of many vessels, some of them contain cells in the lumen (Fig. 5F). Analysis of the CAM from DOX group shows that the mesoderm demonstrates numerous vessels (arrowhead) surrounding a capsulated nested sheet of cells (star). The capsule-like structure (Cp) is formed of fibroblasts and inflammatory cells (Fig. 5F). The generated blood vessels showed a significant increase in the number of the newly formed ones in the DOX group as compared to their counterparts in the CTRL group (Fig. 5C).

Our analysis of CD31 expression, a marker for endothelial progenitor cells, on the CAM tissue, revealed the positive recruitment of endothelial progenitor cells to surround SH-SY5Y cells pre-treated with doxorubicin, facilitating the observed blood vessel formation (Fig. 5G and D).

Examination of the direct effects of doxorubicin on vascular cells using the CAM model. CAMs were treated with doxorubicin (1 µg/ml), and no cytotoxicity was observed in CAM cells, as shown in Supplementary File 1, Fig. 4. At day 7, CAM tissues were harvested, weighed (300 mg per sample), and divided into three groups: control (Ctrl), DOX (1 µg/ml), and DOX (2 µg/ml). An MTT assay revealed a significant increase in cellular viability in the DOX (1 µg/ml) group, while a decrease in viability was observed in the DOX (2 µg/ml) group (Supplementary File 1, Fig. 4).

### The PHD-2/HIF1-α axis is a potential target for doxorubicin to mediate SH-SY5Y cell angiogenesis

**Fig. 6:**
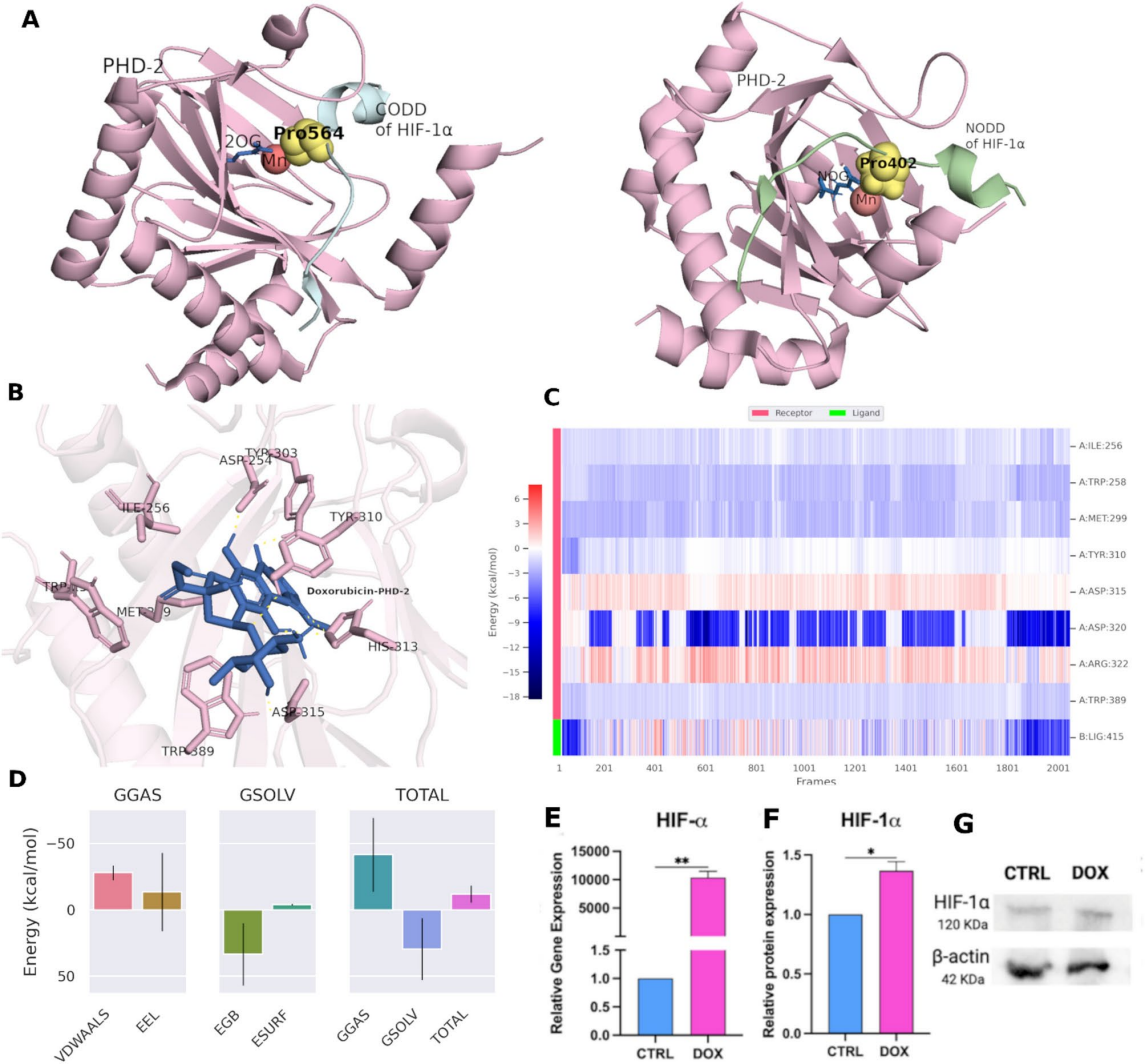
Validating doxorubicin as an inhibitor for PHD-2 to halt HIF1-α degradation. (**A**) (Left panel) CODD domain of HIF-1α in blue color containing proline 564 co-crystallized with PHD-2 (Light pink). (Right panel) HIF-1α NODD domain (green) with proline 402. (**B**) 3D interactions between doxorubicin and the active pocket of PHD-2. (**C**) Heatmap showing the energy contribution of individual residues plotted against time. (**D**) Binding free energy components of the protein–ligand complex. (**E**) Genotypic expression of HIF1-α. (**F**) HIF1-α relative protein expression. G. Western blot bands for HIF1-α and β-actin. *p*-values: * *p* < 0.05, ** *p* < 0.01, *** *p* < 0.001 and **** *p* < 0.0001.

Doxorubicin-treated cells were shown to have an upregulated HIF-1α^36^, we thus investigated the possible structural mechanism of doxorubicin for the upregulation of HIF-1α. Figure 6A illustrates the interaction between PHD-2 (Light pink) and prolyl-residues located in its N/C-terminal oxygen-dependent degradation domains (NODD and CODD). The hydroxylation occurred at the proline 402 residues of HIF-1α of NODD (PDB ID: 5L9B) shown in green, and proline564 of CODD (PDB ID: 5L9V) shown in cyan. Structurally, PHD-2 consists of the catalytic site domain (185–407), C-terminal (400–407), and β2β3-loops (237–254).

To determine the binding affinity of doxorubicin to the co-crystallized structure of EGLN1 with a biologically active inhibitor (PDB code: 5OX6), we first validated our docking protocol using AutoDock Vina, re-docked the co-crystallized inhibitor, Vadadustat, and then carried out a structural comparison which revealed a comparable pose as shown in Supplementary File 1, Fig. 1. Subsequently, doxorubicin scored − 8.5 and adopted pose enabled hydrogen bond formation with the important amino acids of the enzyme’s pocket: Asp315, His313, Tyr303, and Tyr 310 (Fig. 6B).

The docked doxorubicin and PHD-2 were subjected to 100 ns molecular dynamics (MD) simulation using Gromacs for testing their stability in the binding sites in a time-dependent manner^38^.

This was followed by binding free energy calculation to estimate the overall binding strength.

The system of PHD-2-doxorubicin yielded total binding free energy (ΔG) of − 11.98 (Kcal/mol) these free energies are calculated based on the main two terms; the Gibbs free energy of the gas (ΔG_GAS _= − 41.5 kcal/mol) and solvation free energy (Gsolv = 29.53 kcal/mol). The first term ΔG_GAS_ is composed of the sum of electrostatic energy (Δ_ELE_ = − 13.54 kcal/mol) and van der Waals energy (_VDWAALS_ = − 27.96 kcal/mol) (Fig. 6D). Interestingly, heatmap analysis confirmed the maintenance of the energy contribution of individual residues through the simulation time (Fig. 6C).

We further validated the blockage of PHD-2 by studying the gene expression of HIF1-α and degradation blockage at protein levels under normoxic conditions. Our analyses revealed that HIF1-α genotypic expression was upregulated in SH-SY5Y in the DOX group as compared to CTRL (Fig. 6E).

HIF1-α expression and stability were confirmed at the protein level using Western blotting. In SH-SY5Y of the DOX group (treatment at a concentration of 1 µg/ml), HIF1-α protein expression was upregulated compared to their counterparts in the CTRL group (Fig. 6F and G) (Uncropped Western blots: Supplementary File 1, Fig. 3).

HIF1-α activation was further confirmed by the enhancement in SH-SY5Y glycolytic metabolism (Fig. 8). There was an elevated glucose uptake in the conditioned medium of SH-SY5Y and a reduction in H_2_O_2_ production, meanwhile, the cellular pyruvate and lactate production were not altered (Supplementary File 1, Fig. 2).

### Doxorubicin-induced angiogenesis could be a survival mechanism for SH-SY5Y

**Fig. 7:**
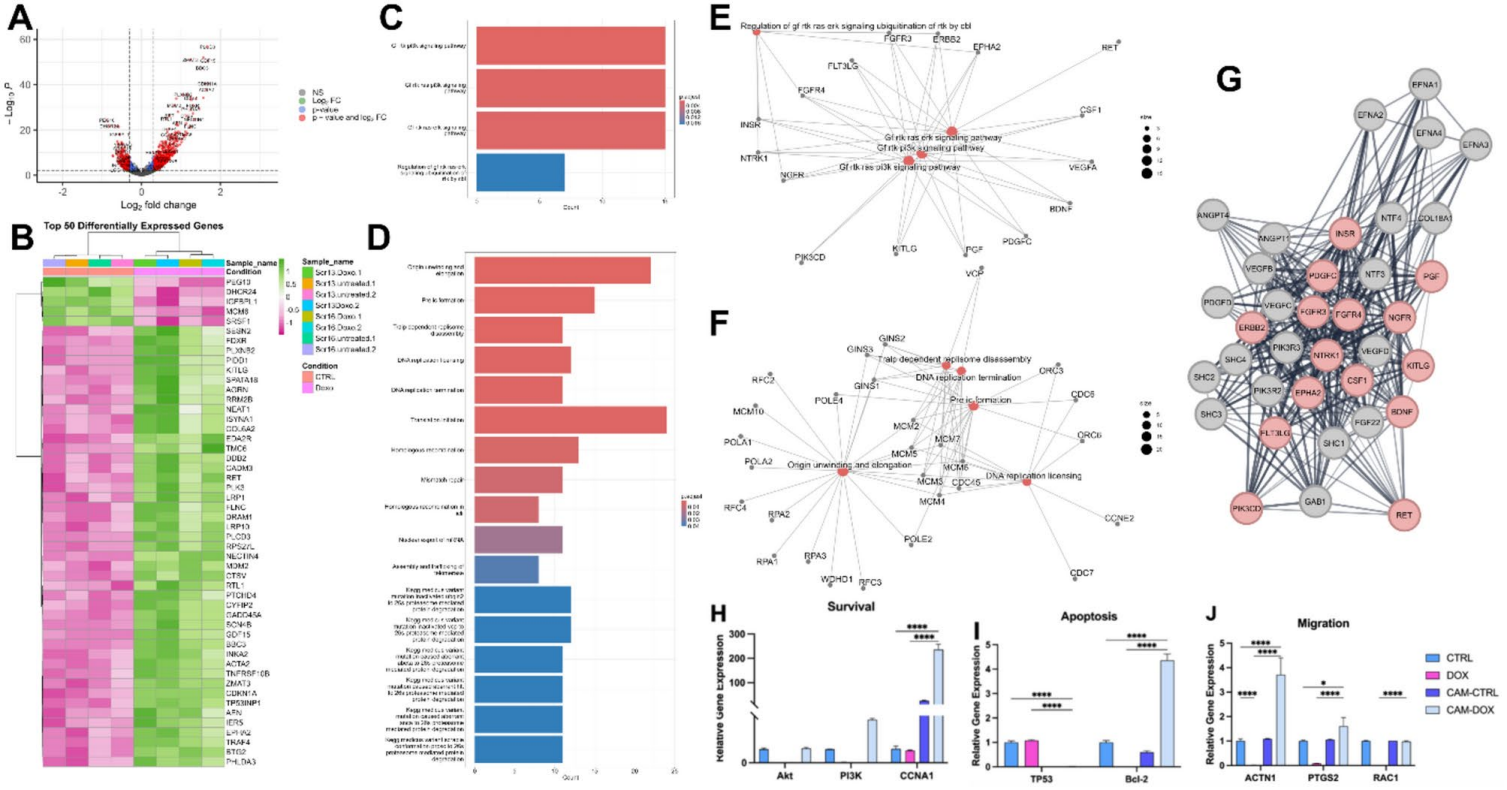
RNA-Seq data analysis of doxorubicin treated SH-SY5Y reveals upregulation of PI3K survival mechanism. (**A**) Volcano plot shows the highly expressed genes in SH-SY5Y DOX-treated compared to CTRL group (red dots represent the upregulated or downregulated genes). (**B**) Heatmap showing the top 50 differentially expressed genes (DEGs) between the doxorubicin-treated SH-SY5Y cells and CTRL group (pink represents downregulated and green represents upregulated genes). (**C**) Bar plot showing pathway enrichment analysis using upregulated DEGs. (**E**) Cnetplot showing pathway enrichment analysis using upregulated DEGs. (**D**) Bar plot showing pathway enrichment analysis using downregulated DEGs. (**F**) Cnetplot showing pathway enrichment analysis using downregulated DEGs. (**G**) Protein–protein interaction network shows 36 nodes interacting with 300 edges (red-colored nodes represent proteins retrieved from the pathway enrichment analysis). (**H**) Genotypic expression of survival-associated markers. (**I**) Gene expression of apoptotic markers. (**J**) Gene expression of cellular migration-related markers. *p*-values: * *p* < 0.05, ** *p* < 0.01, *** *p* < 0.001 and **** *p* < 0.0001.

The volcano plot of the doxorubicin-treated SH-SY5Y cells (at a concentration of 1 µg/ml) and CTRL shows the upregulated genes (on the right) and downregulated (on the left) (Fig. 7A). DEGs analysis shows that most genes have a role in proliferation, cell cycle, or apoptosis processes. There are 849 upregulated DEGs, including PLCD3, KITLG, GDF15, BBC3, and CDKN1A. On the other hand, there were 450 downregulated DEGs, including PEG10, MCM6, and IGFBPL1 which are also represented in the heatmap of the top 50 significant genes (whether up- or down-regulated) (Fig. 7B).

The enriched pathways upon the doxorubicin treatment (at a concentration of 1 µg/ml) are 4 pathways for over-expressed DEGs and 17 for repressed DEGs. Of note, GF/ RTK/Pi3K, GF/ RTK/Ras and GF/RTK/Ras/ERK-mediated survival pathways are enriched from upregulated genes (Fig. 7C, E) whereas origin unwinding and elongation, DNA replication, translation, and repair-related pathways were highly enriched from the downregulated genes (Fig. 7D, F). The PPI network shows the interaction of the upregulated genes implicated in the enriched pathways (Red colored nodes) (Fig. 7G) with each other and with other external proteins from the String database (Grey colored nodes). Importantly, ERBB2, FGFR3, NTRK1, and FGFR4 ranked among the top 10 proteins according to degree centrality.

We then aimed to answer whether this angiogenic response could rescue SH-SY5Y, considering the upregulated Pi3K survival pathway in the DOX-treated cells. Thus, we used the same in-ovo model to culture SH-SY5Y cells pre-treated with DOX (CAM-DOX) and untreated cells (CAM-CTRL) for 7 days and the genotypic expression of the survival, apoptosis, and migration-associated genes.

For cell survival, there was no significant difference in Akt or Pi3K genotypic expression in SH-SY5Y CAM-DOX and those of the DOX and CTRL group (Fig. 7H). CCNA1 was significantly upregulated in SH-SY5Y CAM-DOX as compared to their counterparts in the DOX and CTRL group (Fig. 7H). The genotypic expression of the main apoptosis pathway regulators, P53 and Bcl-2 were assessed. Our data shows significant downregulation of TP53 in the CAM-DOX SH-SY5Y compared to their counterparts in the DOX or the CTRL group (Fig. 7I). As for the anti-apoptotic regulator, Bcl-2, there was a significant upregulation in the CAM-DOX SH-SY5Y when compared to their counterparts in the DOX or the CTRL group (Fig. 7I). We finally aimed to study the genes implicated in cancer cell migration as a mechanism of rescue, thus we analyzed the expression of ACTN1, PTGS2, and RAC1.

## Discussion

The tumor microenvironment is a complex system composed of various cell types, including immune cells, fibroblasts, and endothelial cells, embedded in a matrix of extracellular matrices^39^. Malignant cells can manipulate this complex environment to create conditions that favor their survival, proliferation, and spread^40,41^. Angiogenesis is especially crucial to tumor progression and metastasis by providing nutrition and oxygen supply^42,43^. The oxygen pressure within solid tumors varies, ranging from approximately 5% O_2_ in well-vascularized regions to anoxia near necrotic regions, but is on average in the hypoxic range (about 1% O_2_)^44,45^. In response to hypoxia, tumor cells adapt by changing the transcription of genes involved in angiogenesis, cell survival, and metabolism. The hypoxia-inducible transcription factors HIF-1α and HIF-2α are critical for this adaptive response^46^. Recent research indicates that various cancer modalities, including radiotherapy, photodynamic therapy, and hyperthermia, can elevate HIF-1α levels within tumor cells^47–50^. While research has established that factors beyond hypoxia can stabilize HIF-1α, the influence of chemotherapies on HIF-1α levels within normoxic cancer cells remains largely unknown^51^. HIF-1α could thus have a critical role in cancer resistance to chemotherapeutics and aggressiveness and in understanding its regulation in normoxic environments^52^.

The majority of chemotherapeutic agents, especially doxorubicin, exhibit greater cytotoxicity towards normoxic tumor cells compared to their hypoxic counterparts^53^. This differential sensitivity arises from multiple factors including restricted drug diffusion within hypoxic tumor regions, reduced proliferation rates of hypoxic tumor cells, and enhanced detoxification mechanisms in this hypoxic environment^46^. This paradigm is contingent upon the exposure of normoxic tumor cells to sufficiently high drug concentrations to overwhelm cellular homeostatic, survival, and anti-apoptotic signaling. In the absence of such a cytotoxic threshold, even normoxic tumor microenvironments may harbor drug-resistant cells, particularly those that have induced survival factors such as HIF-1^54^.

In the present study, we showed that doxorubicin treatment to SH-SY5Y NB cells reduced their proliferative capacity (Fig. 1) and induced their apoptosis (Fig. 2) under normoxic conditions, as reported previously^55,56^. Yet, doxorubicin treatment upregulated the expression of the pro-angiogenic factors, VEGF and PDGF, in addition to induction of NO secretion, a biochemical molecule crucial for tumor angiogenesis (Fig. 3)^57–60^. We established an indirect co-culture system between SH-SY5Y pretreated with doxorubicin to confirm in-vitro HUVECs vasculogenic response (Fig. 4) as described in^61^. We also used a direct in-ovo system to further confirm the neo-angiogenesis of SH-SY5Y on chick embryos’ CAM (Fig. 5), both of which showed positive results. Previously, tumor cells were reported to stimulate neo-angiogenesis through the production of growth factors like VEGF-A and bFGF, which recruit and activate endothelial progenitor cells. These cells contribute to new blood vessel formation, while tumor-induced hypoxia facilitates angiogenesis and metastasis by promoting ECM degradation and vessel growth^62^. Our analysis of CD31 expression confirmed the recruitment of endothelial progenitor cells to the SH-SY5Y cells. A comparative analysis of samples from pre- and post-chemotherapy treated tumors, collected from high-risk NB patients revealed a consistent increase in vessel density following treatment. Despite evidence of necrotic areas indicating therapeutic efficacy, patient outcomes varied: three achieved event-free survival, while four experienced relapses^37^. Moreover, chemotherapy-induced mobilization of bone marrow-derived circulating endothelial progenitor cells^63,64^.

**Fig. 8:**
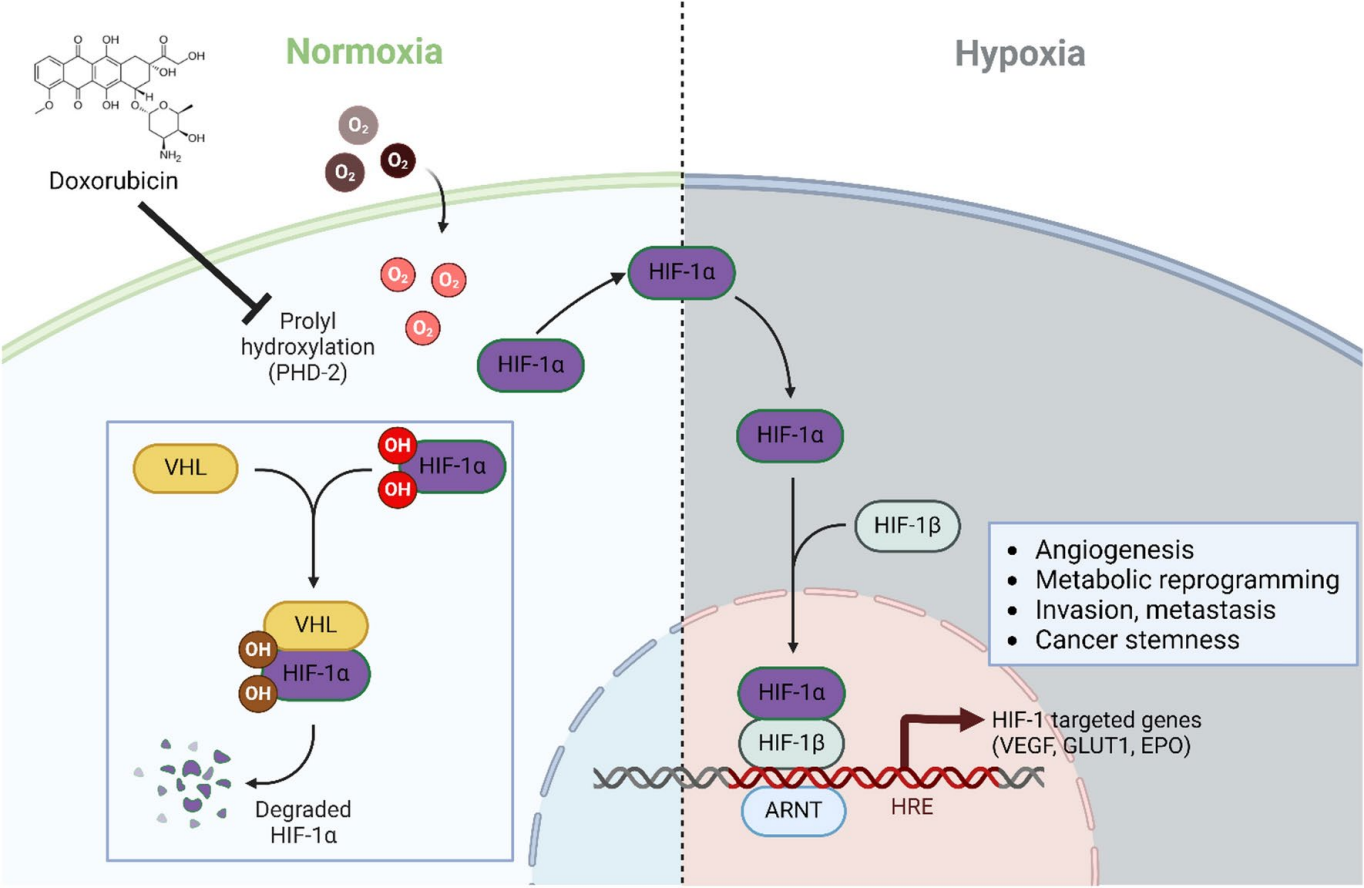
A schematic representation of the proposed mechanism via which doxorubicin induces HIF-1α downstream cascades in SH-SY5Y NB cells.

Previously, it was reported that doxorubicin treatment upregulates the HIF-1α expression and activity^36^, which could be promoting the angiogenic program expression. HIF-1α is a heterodimeric transcription factor that regulates the response to hypoxia via upregulation of an array of genes^65^. The transcriptional activity of HIF-1α is oxygen-dependent and regulated through post-transcriptional hydroxylation of HIF-a domains^23,66^. Accordingly, during hypoxia factor inhibiting HIF (FIH) and oxoglutarate (2OG) consolidate the hydroxylation of an asparagine-residue in the C-terminal transcriptional activation domain (CTAD) of HIF1-α^67^. However, under normoxic conditions, two domains of HIF-1α are subjected to hydroxylation via prolyl hydroxylases (PHDs or EGLNs) that increase the susceptibility to proteasomal degradation via the targeting component of E3 ubiquitin ligase system which is Von Hippel-Lindau protein (pVHL) since it has higher binding affinity to hydroxylated domains than the unmodified residues^68,69^. The PHD family includes three members (PHD1, PHD2, and PHD3), however, PHD2 also known as (EGLN1) is the essential regulator of HIF-1α, considering it a propitious target for HIF-1α stabilization^70^. Thus, PHD-2 inhibitors designed as HIF-1α stabilizers achieved clinical efficacy in the treatment of anemia and ischemic and have been linked to cancer treatment-associated diseases^71–74^. Additionally, recent studies are implementing this strategy for searching for HIF-1α pathway activators^75,76^. Thus, our analyses were designed to investigate the possible blocking of PHD-2 activity by doxorubicin, promoting HIF1-α stability and expression. Doxorubicin demonstrated the ability to inhibit PHD-2 by interacting with the same amino acid residues as the co-crystallized inhibitor, Vadadustat. This binding was confirmed through molecular docking, molecular dynamics simulations, and binding free energy calculations (Fig. 6). Further, this was confirmed by the upregulated genotypic expression of HIF1-α and HIF1-α protein non-degradation and stability in-vitro under normoxic conditions (Fig. 6E–G). This upregulation of HIF1-α reveals that surviving normoxic tumor cells can accumulate HIF-1α, promoting VEGF secretion after doxorubicin treatment and their neo-angiogenesis, which could serve as a survival mechanism (Fig. 8).

To determine whether angiogenic response could act as a survival mechanism, we used RNA-Seq data for SH-SY5Y cells treated with doxorubicin and untreated and analyzed the differentially expressed genes followed by pathway enrichment analysis. Of the upregulated DEGs, PLCD3 was previously reported to be implicated in the proliferation, migration, and invasion of nasopharyngeal carcinoma cells^77^. KITLG is an upregulated DEG and is reported to mediate innate resistance to the chemotherapeutics displayed by glioblastoma cells^78^. Moreover, GDF15 was reported to be implicated in protecting mitochondrial function and inhibiting apoptosis in SH-SY5Y cells after exposure to rotenone by upregulating PGC1α via p53^79^. These properties might comprise its anti-apoptotic effects, mediated by the PI3K/Akt/mTOR signaling pathway^79^. On the other hand, there were 450 downregulated DEGs, including PEG10, whose silencing inhibits the progression of neuroblastoma by regulating the microRNA-449a (miR-449a)/ribosomal protein S2 (RPS2) axis^80^. Minichromosome maintenance complex component 6 (MCM6) is the driving force of G1/S cell cycle progression, and it is also a prognostic marker in NB^81^. It was found to be downregulated in doxorubicin-treated SH-SY5Y (Fig. 7), confirming the halted cell cycle progression.

Of interest, the upregulated pathways in SH-SY5Y doxorubicin-treated cells included the GF/RTK/Pi3K signaling pathway, GF/RAS/ERK ubiquitination of RTK signaling cascade, and GF/RTK/RAS/Pi3K pathway activation (Fig. 7). These pathways are well known for their involvement in cancer cell survival^41,82,83^. The expression of the genes involved in those pathways was also validated in-vitro upon culture of SH-SY5Y cells on CAM for neo-angiogenesis. For instance, Bcl-2, an anti-apoptotic marker^84,85^ was found to be upregulated in the CAM-DOX SH-SY5Y cells when compared to doxorubicin-treated SH-SY5Y cells. Contrarily, TP53 was found to be repressed, which further aided their survival from apoptosis^86^. Specifically, there was no significant difference between the expression of Akt and Pi3K in CAM-DOX SH-SY5Y cells and the CTRL group, evident for their survival. Also, other genes (PTGS2, RAC1, and ACTN1) that are involved in tumor metastasis were found to be significantly upregulated in the CAM-DOX SH-SY5Y cells as compared to CTRL, which could be implicated in their mobilization and metastasis.

It was reported that doxorubicin-induced Akt activation is a key mediator of NB cell survival. Akt inhibitors enhance NB cell sensitivity to doxorubicin by suppressing various pro-survival mechanisms^87–91^. Akt inhibits pro-apoptotic signaling, including Fas ligand expression and BAD phosphorylation, leading to Bcl-xL release, the inhibition of pro-apoptotic caspase-9, and glycogen synthase kinase 3-β^87^. Additionally, Akt modulates signaling pathways involved in apoptosis and stress responses such as ZAK/MKKK4/MKKK7/JNK module^89,91^. Doxorubicin-resistant NB cells secrete paracrine factors that activate pro-survival signaling in neighboring cells^89,91,92^. NB cells also exhibit doxorubicin-induced pro-survival Src signaling associated with impaired p53 signaling^93^. Histone deacetylase and JAK2 inhibitors can reduce doxorubicin resistance by targeting epigenetic modifications and signaling pathways^94,95^. Both doxorubicin resistance and doxorubicin-induced senescence can enhance tumor cell stemness and dormancy, increasing the risk of aggressive post-therapeutic relapse^96^. Understanding these mechanisms is crucial for developing effective treatments for NB.

Overall, while our study provides significant insights into the role of doxorubicin in blocking PHD-2 to stabilize HIF-1α and inducing angiogenesis, several limitations should be acknowledged to ensure transparency and guide future research. First, our experiments primarily focused on a single cancer type, neuroblastoma, using the SH-SY5Y cell line as a representative model. While this approach enabled detailed mechanistic exploration, it limits the generalizability of our findings to other cancer types. Additionally, we employed an in-ovo model for angiogenesis, which, while effective for assessing neo-angiogenic responses, does not fully replicate the complexity of in-vivo tumor environments. Future studies should expand upon our findings by incorporating multiple neuroblastoma cell lines, additional cancer types, and advanced in-vivo models to validate the broader applicability and reproducibility of our results. Further research should be done to explore the role of doxorubicin in promoting SH-SY5Y migratory response and metastasis through angiogenesis. These steps will help further strengthen the translational potential of our study.

## Conclusions

In conclusion, doxorubicin, a common NB therapy, effectively reduced SH-SY5Y cell proliferation and triggered apoptosis. However, it unexpectedly induced pro-angiogenic factors secretion. Co-cultured HUVECs with doxorubicin-treated SH-SY5Y cells displayed enhanced proliferation, migration, and tube formation. In-ovo studies confirmed the doxorubicin effect on SH-SY5Y NB cells’ neo-angiogenic activity. Mechanistically, PHD-2 inhibition and Pi3K pathway activation might be involved. These findings reveal a paradoxical pro-angiogenic response and suggest targeting PHD2 and the Pi3K pathway for a more comprehensive NB treatment approach.

## Material and methods

### Cell culture

SH-SY5Y NB cell line (a gift from the Department of Pathology, Children’s Cancer Hospital 57,357, Egypt) was maintained in complete culture medium (CCM) consisting of RPMI 1640 medium (PAN Biotech, Germany) and DMEM/F12 (Gibco, MA, USA) 1:1 supplemented with 10% heat-inactivated fetal bovine serum (FBS) (BioWest, USA), 1% non-essential amino acids (Lonza, Switerland), 1% streptomycin/penicillin (PAN Biotech, Germany) and 1% L-glutamine (PAN Biotech, Germany). Human umbilical vein endothelial cells (HUVEC) (Thermo Fisher Scientific, USA) were cultured in DMEM/F12 (Biowest, France) supplemented with 2% FBS, 2% penicillin/streptomycin/amphotericin B, 2% L-glutamine, 1 µg/ml dexamethasone (Amrya, Egypt), 250 ng/ml insulin (Acros Organics, USA), 20 µg/ml heparin (Nile, Egypt), 0.025 µg/ml ascorbic acid (Sigma-Aldrich, USA), 5 ng/ml epidermal growth factor (EGF) (Pepro Tech, UK), and 10 ng/ml basic fibroblast growth factor (b-FGF) (Pepro Tech, UK). All cells were incubated at 37˚ C in a humidified 5% CO2 incubator.

SH-SY5Y was cultured in CCM (CTRL), or DOX: CCM containing 1 µg/ml doxorubicin for 24 h (Experimental group).

### Flow cytometric cell cycle analysis

SH-SY5Y cells were detached from their culture plates, collected by centrifugation, and washed with phosphate buffer solution (PBS), and fixed with 75% ethanol at − 20 °C. For the cell cycle analysis, the fixed cells were washed with PBS and treated with ribonuclease A to remove RNA, which can interfere with DNA staining. Propidium iodide (PI) stain was then added to the cells and incubated at 4 °C for 30 min. Finally, the cells were resuspended in PBS and analyzed using a flow cytometer (FACSCalibur) to measure the amount of PI stain in each cell, which corresponds to its DNA content and thus cell cycle stage. Standard analysis protocols were followed throughout this process using specific software (CellQuest Pro), and the data was further analyzed using FlowJo software.

### Real-time quantitative PCR (RT-qPCR)

Total RNA was extracted from SH-SY5Y in the CTRL, DOX, CAM-CTRL, and CAM-DOX using TriQuick Reagent (Solarbio® Life Sciences, Beijing, China). Purity and concentration were quantified using a NanoDropTM 2000/2000c spectrophotometer (ThermoScientific, Waltham, MA, USA). Followed by cDNA synthesis using RevertAid First StrandcDNA Synthesis Kit (Thermo Fisher) following manufacturer protocol. Final PCR was quantified using the cDNA samples using HERA PLUS SYBR® Green kit (Willowfort, UK) according to the manufacturer’s protocol. Genes for apoptosis, proliferation, and angiogenesis were detected (Supplementary File 1, Tabel 1), and GAPDH was used as a housekeeping gene. Reactions were carried out using the CFX96 Touch™ Real-Time PCR Detection System.

### Apoptosis analysis

Induction of cellular apoptosis by the doxorubicin-treated SH-SY5Y NB cells was analyzed by flow cytometry using Annexin V FITC and PI apoptosis kit (Miltenyi Biotec Inc., Auburn, CA, USA) as per the manufacturer’s protocol.

### MTT proliferation assay

SH-SY5Y cells were seeded at a concentration of 5000 cells/well in 96 well plates in both CTRL and DOX groups. SH-SY5Y from CTRL group and DOX group (pretreated with DOX) indirectly co-cultured with HUVECs (seeded at a concentration of 10,000 cells/well of 6-well plates) for 48 h. Following the treatment or co-culture, the MTT reagent 3-(4,5-dimethylthiazol-2-yl)-2,5-diphenyltetrazolium bromide (Life Technologies, USA) was added to each well containing SH-SY5Y or HUVECs at a final concentration of 5 mg/ml, and incubated for 3 h in a 5% CO2 humidified incubator at 37 °C as done previously^97,98^. The formed formazan salts were dissolved in anhydrous dimethyl sulfoxide (DMSO) for 15 min on a shaking plate. The optical density (OD) was quantified at 570 nm with reference to 630 nm using a FLUOstar Omega-microplate reader (BMG Labtech, Cary, NC, USA).

### HUVEC migration assay

HUVEC were cultured in a 24-well plate Transwell system (ThinCert cell culture insert, sterile, translucent polyester filter membrane, 8-µm pore diameter; Greiner, Germany) with a seeding density of 3000 cells/well. Briefly, cells were suspended in 200 µL FBS-free medium and seeded into the upper compartments. 500 µl complete medium was added into the lower compartments supplemented with 20% FBS, after which the cells were incubated at 37 °C for 48 h. Migrating cells on the lower surface of the filter were fixed in 4% paraformaldehyde and stained with 0.5% crystal violet. Cells on the upper surface of the filter were carefully removed using a cotton swab. Stained cells were counted under a Leica Dmi8 inverted fluorescent microscope (Leica Microsystems, Wetzlar, Germany), and analyzed using the ImageJ software (NIH, USA) program.

### In-vitro vasculogenesis using tube formation assay

HUVECs (5000 cells/well) were cultured on a collagen basement membrane matrix (Millipore, USA) and diluted in HUVEC CCM^38^. The matrix was coated on 6-well plates at 37 °C for 1 h. SH-SY5Y cells (5000 cells/well) from either the CTRL or DOX group were then co-cultured with an equal number of HUVECs in the same well. After 24 h of incubation, the tube formation ability of HUVECs was assessed by quantifying the number of tubes and nodes within the formed network.

### Enzyme-linked immunosorbent assay (ELISA)

Intracellular vascular endothelial growth factor A (VEGF-A) levels were measured using a commercially available ELISA kit (Elabscience, USA, Cat No. E-EL-H0111). Following a standard ELISA protocol, as done previously^97,98^, samples or standard solutions were added to wells on a pre-coated plate containing antibodies specific to VEGF-A. Following incubation and washing, a biotinylated detection antibody binds specifically to captured VEGF-A. An enzyme conjugate, Avidin-Horseradish Peroxidase (HRP), was then added, and cells were incubated and washed. The reaction was stopped at a defined time, and the resulting color intensity was measured at a wavelength of 450 nm using a plate reader (BMG Labtech, FLUOstar Omega, BMG Labtech, Cary, NC, USA).

### Analysis of biochemical reprogramming in SH-SY5Y and HUVECs

Biochemical alterations were assessed to investigate the effects of DOX treatment on SH-SY5Y cells and when co-cultured with HUVECs. The following parameters were measured in the conditioned medium: glucose consumption, lactate production, hydrogen peroxide (H_2_O_2_) production, and NO production. Specific enzymatic colorimetric assay kits from Biodiagnostic (Cairo, Egypt) were used for glucose and H_2_O_2_, while kits from Spectrum Diagnostics (Cairo, Egypt) were used for lactate (catalog number 274 001). NO production was measured using a Biodiagnostic kit. All assays were performed according to the manufacturer’s instructions. Intracellular pyruvate levels in SH-SY5Y cells were quantified using a commercially available pyruvate (pyruvic acid) quantitative UV kit (GL1320UPL, Diagnostic, Mainz, Germany).

### Immunocytochemistry analysis

To investigate the contractility of HUVECs upon co-culture with SH-SY5Y NB cells, cells were seeded onto glass slides, co-cultured with SH-SY5Y cells in CTRL and DOX groups for 24 h, washed, and fixed with 4% paraformaldehyde. Cells were then permeabilized with 1% Triton-X 100 and incubated with 1% bovine serum albumin (BSA) solution to block nonspecific binding of antibodies. Primary mouse α-smooth muscle actin (α-SMA) (Invitrogen, USA) was used with secondary goat anti-mouse (Molecular Probes, USA) to stain cellular α-SMA. Nuclei were counterstained with Hoechst 33,342 (Molecular Probes, USA) for 15 min. Cells were visualized using a Leica DMi8 inverted fluorescent microscope (Leica Microsystems, Wetzlar, Germany).

### Western blot

SH-SY5Y cells from both CTRL and DOX groups were collected, and the cell pellet lysed in ice-cold lysis buffer (150 mM NaCl, 50 mM Tris HCl, and 1% Triton X-100, at pH = 8) after adding protease inhibitor cocktail (Thermofisher Scientific) to a final concentration of 10%. Protein quantification was performed using a Bradford reagent. The proteins were mixed with SDS sample buffer and incubated at 95 °C for 5 min. After electrophoresis, proteins from each group were transferred to the polyvinylidene fluoride membranes (Bio-Rad Laboratories Inc., Hercules, CA, USA). Primary anti-human HIF1-Alpha antibody (Cell Signaling Technology, USA) and anti-human beta-actin (Novus Biologicals, USA) were added after blocking with 5% nonfat milk for 1 h. at 4 °C, and membranes were incubated overnight at 4 °C. The membranes were then treated with goat anti-mouse IgG (H + L)-HRP conjugate or goat anti-rabbit IgG (H + L)-HRP conjugated secondary antibodies (Bio-Rad, USA) for 1 h at 4 °C. Finally, the chemiluminescent signals were detected using an enhanced chemiluminescence solution, clarity™ Western ECL blotting substrate (Millipore, USA). The protein band signals on the membranes were visualized using the ChemiDoc™ MP Imaging System (Bio-Rad, USA). Full uncropped western blot is shown in Supplementary File 1, Fig. 3.

### Chick embryo chrioallantoic membrane (CAM) angiogenesis assay

Pathogen-free fertilized Egyptian Fayoumi eggs (Poultry Center, El-Azab, El-Fayoum, Egypt) at early embryonic stage were incubated vertically in an auto-rotator 37^o^ C egg incubator till reaching 7 days of embryonic development (7.5 E stage). A window was opened under sterile conditions using a mini-drill at the stamped area of the egg where the air-sac is located to uncover the air-sac membrane that covers the CAM area. Afterward, the air-sac membrane was removed carefully using sterile forceps to reveal the blood vessel network of CAM. The opened window was sealed with stretch-film and the eggs were incubated in a stable egg-rack inside the incubator pending inoculation.

To assess the changes in the angiogenic potential of SH-SY5Y cells from CTRL and DOX, a cell suspension was embedded in a collagen matrix to clump the cells together and compared to the Sham group (no cell inoculation). Then, 50 µl of the mixture was implanted between two major veins of CAM in the CTRL and DOX groups. The eggs were left for 5 min till the solidification and settlement of the implanted cells on the CAM surface and the eggs were sealed with stretch film and incubated for 7 days.

On day 14, the number of generated blood vessels surrounding the implanted cells was investigated in-ovo and ex-ovo. For isolating CAM, a 6-well plate was prepared with 4 ml of 4% paraformaldehyde (PFA) for fixation, then, the CAM was isolated using sterile scissors and forceps. The CAM was held upward using forceps and a small cut was made at the edges of the membrane, the rest of the membrane was cut using scissors. The CAM then was placed in a well of 6-well plate and the chick embryo was euthanized immediately after CAM isolation.

### Histological analysis of CAM sections

The specimens of the CAM were transferred into 10% formalin solution then trimmed, cleared in xylene, impregnated, and embedded in paraffin. Deparaffinized sections (4 mm thick) were stained with hematoxylin and eosin (H&E) according to the standard protocol.

For quantification of the vessel’s density, the number of the blood vessels was estimated under the light microscope (Leica, Germany) connected to a digital camera (Olympus, Japan) at a magnification of 400× in the H&E-stained sections. The vessels were identified as endothelial-lined spaces, either empty or containing cells. Followed by CD31 (Sigma-Aldrich, USA) immunohistostaining to assess the endothelial progenitors.

### Computational chemistry analyses

#### Molecular docking

The protein structures of PHD-2 were retrieved from the protein data bank (PDB IDs: 2HBT, 5L9B, 5L9V, and 5OX6). The docking studies were carried out using AutoDock Vina (version 1.1.2)^99^, The Python script (prepare receptor4.py) offered by the MGLTools package (version 1.5.4) was used to convert the structures to PDBQT^100^. The search algorithm’s efficiency was retained at its default settings, and the grid box docking dimensions were 22 Å × 23 Å × 22 Å, with a spacing of 1 Å to accommodate all the possible conformations of the docked molecules. All the visualization figures were generated using Pymol software.

#### Molecular dynamics simulation

Molecular dynamics simulation run was conducted using GROMACS 2024.2^101^. The solvation of each protein–ligand complex was carried out in a dodecahedron box of the TIP3P explicit water model^102^. The system was then neutralized using NaCl ions with ionic strength of 0.1 M concentration. The steepest descent minimization algorithm was utilized by a convergence set at 10 kJ/mol and 50,000 steps for system energy minimization. At 300 K temperature and 1 atm pressure, each NVT followed by NPT equilibration was conducted for 500 ps, after a production run at the NPT ensemble was performed for 100 ns.

#### Binding free energy calculation

The binding free energy of doxorubicin was evaluated via the MMGBSA method by using the gmx_MMPBSA tool v1.5.2^103^.

### Computational biology analyses

#### Differentially expressed genes (DEGs) analysis

FASTQ files were retrieved from the GSE197243 study in the Gene Expression Omnibus (GEO) website and processed by the Galaxy public server at usegalaxy.org^104,105^. We selected the two wildtype clones of the SHSY5Y cell line (SHSY5Y-SCR13 and SHSY5Y-SCR16 from this dataset with two conditions: treated with doxorubicin and nontreated cell lines (n = 8). To obtain the row count matrix, quality control, trimming, mapping, gene counting, and annotating were performed by FASTQC^106^, Trimmomatic^107^, HISAT2^108^, featureCounts^109^, and annotateMyIDs^110^, respectively. The row count matrix was analyzed by the DESeq2 package^111^ in R to study the DEGs between SH-SY5Y DOX-treated and nontreated cell lines. For visualization, the volcano plot and heatmap were further generated by EnhancedVolcano and heatmap packages.

#### Pathways enrichment analysis

Pathway enrichment analysis was performed using the clusterProfiler package (version 4.10.1) in R^112^. The KEGG MEDICUS dataset was downloaded from the Molecular Signatures Database (MSigDB)^113–115^ and used to generate the background gene set^116,117^. The upregulated and downregulated genes (*p*-value < 0.05) were separated and the enriched pathways list with *p*-value < 0.05 were produced using enricher function for each. The resulting pathways were represented as barplot and cnetplot to show each list’s significantly enriched pathways.

#### Protein–protein interaction (PPI) network analysis

To visualize the interaction between the upregulated genes involved in the enriched pathways, we use Cytoscape software (version 3.10.2)^118^. 16 genes were introduced to the String plugin and the network was expanded by 20 more interactors with 0.5 selectivity. The degree of centrality was measured using Cytohubba^119^.

### Statistical analysis

Data are presented as the mean ± SD using GraphPad Prism version 10.2.3. An unpaired two-tailed student t-test was used to analyze the statistical significance between 2 groups. For comparisons between more than 2 groups, a one-way ANOVA test was used. *p*-values: * *p* < 0.05, ** *p* < 0.01, *** *p* < 0.001 and **** *p* < 0.0001 were considered statistically significant.

## Supplementary Information

Supplementary Information.

## Data Availability

This study used publicly available datasets for RNA-Seq analysis. Datasets used in RNA-Seq analysis were retrieved from Gene Expression Omnibus (GEO) database under accession numbers GSE197243. The code for reproducing the RNA-Seq data analysis is available in the GitHub repository. See Code: https://github.com/aabousha/Angiogenesis-paper.
