# Supplementary material for: PHD-2/HIF-1α axis mediates doxorubicin-induced angiogenesis in SH-SY5Y neuroblastoma microenvironment: a potential survival mechanism
Source: Sci Rep. 2025 Mar 3;15:7487. doi: 10.1038/s41598-025-89884-3 (PMC11876694; doi:10.1038/s41598-025-89884-3)
Supplement: Supplementary file 1 — Supplementary Information. [file 41598_2025_89884_MOESM1_ESM.docx]

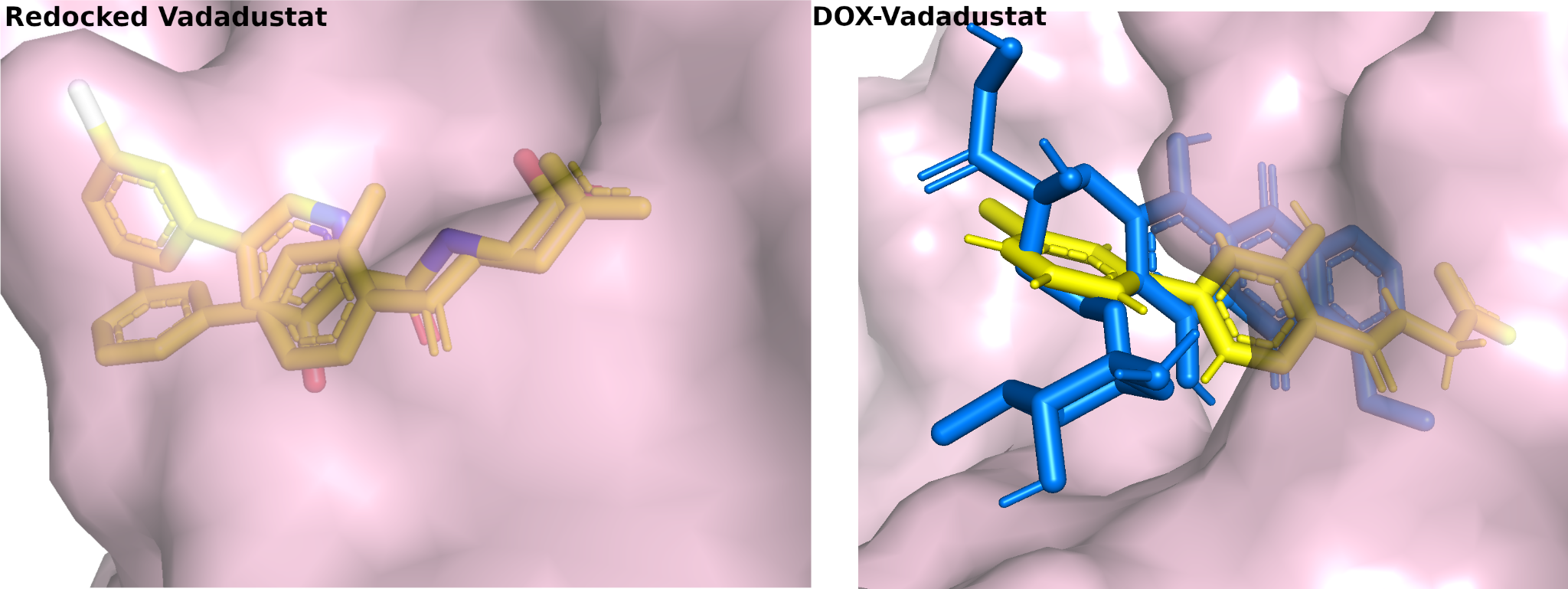


**Fig. 1** **(Left panel)** Re-docked structure of co-crystallized Vadadustat. **(Right panel)** Superimposed Pose of DOX and Vadadustat


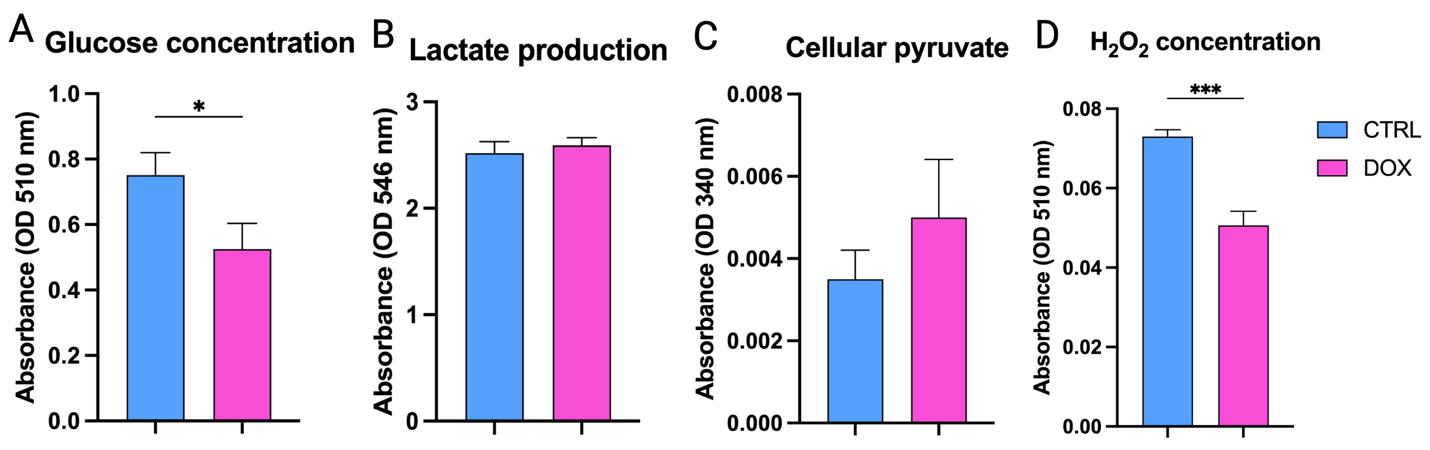


**Fig. 2 Analysis of SH-SY5Y glycolytic metabolism to HIF1-α activation.** A. Glucose concentration in SH-SY5Y conditioned medium. B. Lactate production. C. Intracellular pyruvate concentration. D. H_2_O_2_ quantification.


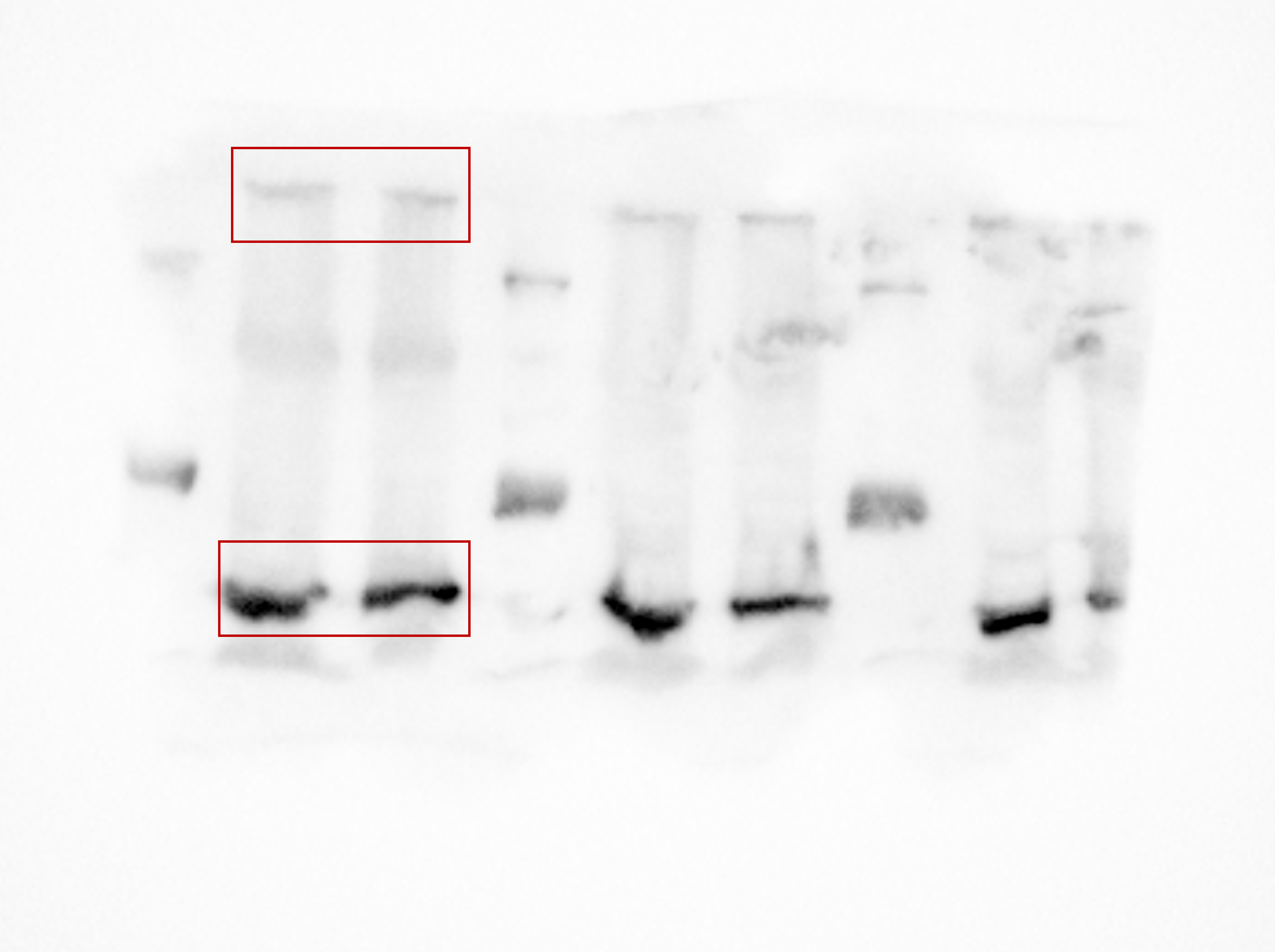


Fig. 3 Uncropped western blot for beta-actin and HIF-alpha


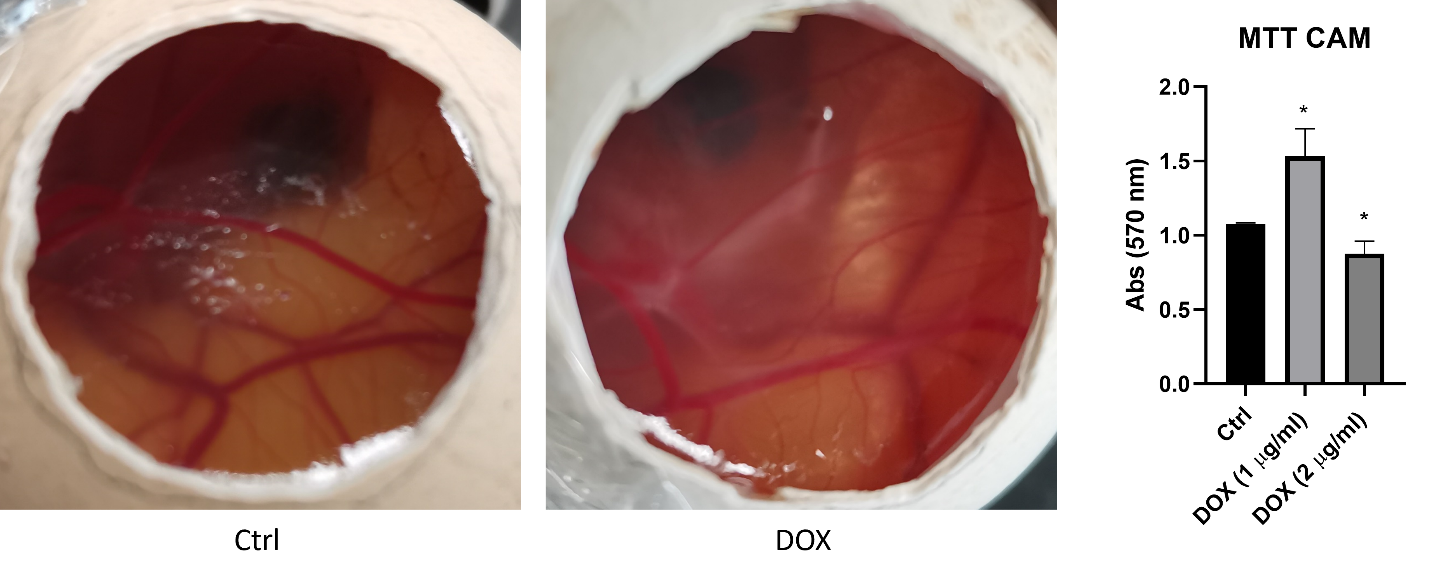


Fig. 4 Effect of doxorubicin on CAM vascular cells.


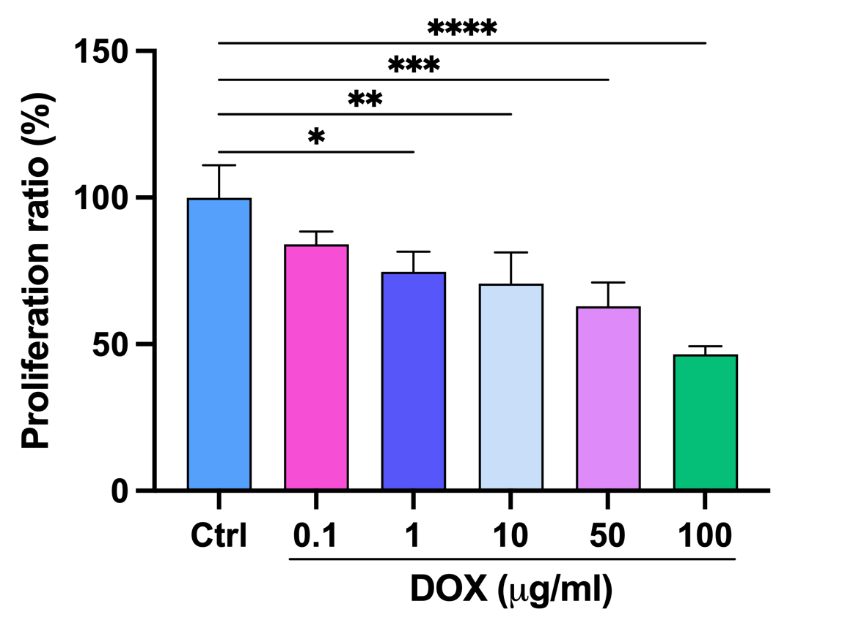


Fig. 5: SH-SY5Y proliferation rate upon doxorubicin treatment at 0.1, 1, 10, 50, and 100 µg/ml concentrations.

**Table 1**: RT-qPCR primer sequences.

| Gene name | Forward primer (5’-3’) | Reverse primer (3’-5’) |
| --- | --- | --- |
| GAPDH | GGTGAAGGTCGGAGTCAACG | CAAAGTTGTCATGGATGACC |
| c-MYC | GCGACTCTGAGGAGGAA | TGCGTAGTTGTGCTGATG |
| n-MYC | CGCAAAAGCCACCTCTCATTA | TCCAGCAGATGCCACATAAGG |
| PHOX2B | TCAGGGACCACCAGAGCAGT | GGTGAAAGTGGTGCGGATGC |
| hTERT | CGGAAGAGTGTCTGGAGCAA | GGATGAAGCGGAGTCTGGA |
| TOP1 | GGCACTGGTATCCTGAAAAGCC | GTGGCAGATTGCCACGAACAT |
| P21 | GATGGCACCAGAGGTGGTTA | TCCCGAAATATTGGGGAAAG |
| BAX | CAAACTGGTGCTCAAGGCCC | GAGACAGGGACATCAGTCGC |
| Caspase 3 | ACAGTGGAACTGACGATGATATG | TCCCTTGAATTTCTCCAGGAATAG |
| P53 | GGAAGAGAATCTCCGCAAGAA | AGCTCTCGGAACATCTCGAAG |
| Bcl-2 | GGATAACGGAGGCTGGGATG | TGACTTCACTTGTGGCCCAG |
| VEGF | ATCTGCATGGTGATGTTGGA | GGGCAGAATCATCACGAAGT |
| PDGF | GATACTTTGCGCGCACACAC | GGTTTTCTCTTTGCAGCGAGG |
| MMP2 | TTGCAGGAGACAAGTTCTGGAG | CGATGAGCTTAGGGAAAC |
| MMP3 | CACTCACAGACCTGACTCGGTT | AAGCAGGATCACAGTTGGCTGG |
| MMP13 | TTCGGCTTAGAGGTGACTGGC | TTCACCCACATCAGGAACCCC |
| HIF-alpha | CGATGAGCTTAGGGAAAC | CGATGAGCTTAGGGAAAC |
| AKT | CAGCTGATGAAGACGGAGCG | GTCTCCACATGGAAGGTGCG |
| Pi3K | CACTGTCCATTGGCATGGGG | GTTACTCAGTCCTGCGTGGG |
| CCNA1 | GCACACTCAAGTCAGACCTGCA | ATCACATCTGTGCCAAGACTGGA |
| ACTN1 | AACTGTCACTTGGCGGGCAGGG | AAGGGCATCAGCCAGGAGCAGAT |
| RAC1 | AAGCTGACTCCCATCACCTATCCG | CGAGGGGCTGAGACATTTACAACA |
| PTGS2 | CCCTTGGGTGTCAAAGGTAA | GCCCTCGCTTATGATCTGTC |
